# Polypyrrole Aerogels: Efficient Adsorbents of Cr(VI) Ions from Aqueous Solutions

**DOI:** 10.3390/gels9070582

**Published:** 2023-07-17

**Authors:** Patrycja Bober, Islam M. Minisy, Zuzana Morávková, Helena Hlídková, Jiří Hodan, Jiřina Hromádková, Udit Acharya

**Affiliations:** Institute of Macromolecular Chemistry, Czech Academy of Sciences, 162 00 Prague, Czech Republic; minisy@imc.cas.cz (I.M.M.); moravkova@imc.cas.cz (Z.M.); hlidkova@imc.cas.cz (H.H.); hodan@imc.cas.cz (J.H.); hromadkova@imc.cas.cz (J.H.); acharya@imc.cas.cz (U.A.)

**Keywords:** polypyrrole, aerogel, conductivity, adsorbent, hexavalent chromium

## Abstract

Three-dimensional and porous polypyrrole (PPy) aerogels were prepared using a facile two-step procedure in which cryogels were synthesized via the cryopolymerization of pyrrole with iron (III) chloride in the presence of supporting water-soluble polymers (poly(*N*-vinylpyrrolidone), poly(vinyl alcohol), gelatin, methylcellulose or hydroxypropylcellulose), followed by freeze-drying to obtain aerogels. The choice of supporting polymers was found to affect the morphology, porosity, electrical conductivity, and mechanical properties of PPy aerogels. PPy aerogels were successfully used as adsorbents to remove toxic Cr(VI) ions from aqueous solutions.

## 1. Introduction

Recently, the rapid growth of the world’s population has led to increased industrialization, which has unfortunately influenced the environment negatively through the contamination of surface- and groundwater with heavy metal ions (Cr(VI), Pb(II), Cd(II), Hg(II), etc.) [1,2,3]. Among these, hexavalent chromium, Cr(VI), is widely produced by various industries, including chrome plating, dye manufacturing, the leather and textile industries, wood preservation, the paint industry, etc. [4,5,6]. Additionally, chromium occurs in groundwater naturally due to the erosion of natural chromium deposits. The World Health Organization has stated that the total amount of chromium in drinking water should not exceed 0.05 mg L^−1^. Due to the harmful nature of Cr(VI) to humans (as a carcinogenic, skin irritant, allergen, etc.) [5,7], many techniques are applied to remove it from water nowadays, for example, electrochemical [8] or catalytic reduction [6], photoreduction [9], ultrafiltration [10,11,12], and adsorption [4,5,7]. 

Among these methods, adsorption has become the most popular owing to its low cost, easy performance, and high efficiency. However, the preparation of adsorbents with good selectivity, stability, and reusability, as well as easy removability from wastewater, is still a challenge [13]. Currently, adsorbents based on conducting polymers have become popular candidates for the removal of wastewater pollutants [4,14]. Polypyrrole (PPy), polyaniline (PANI), poly(3,4-ethylenedioxythiophene) (PEDOT) and poly(p-phenylenediamine) are some of the most common examples of conducting polymers, which, either pristine or as a part of composites, have been widely used for the adsorption and/or photocatalysis of organic anionic and cationic dyes (Reactive Black 5, methyl orange, safranin, etc.), heavy metal ions (Cr(VI), Pb(II), etc.), drugs (tetracycline, diclofenac, etc.), personal care products (e.g., caffeine)**,** etc. from wastewater [4,5,6,7,8,9,13,14,15,16]. Gupta et al. synthesized magnetoconductive PEDOT/maghemite composites and applied them to the removal of an anionic dye, Reactive Black 5, from the aqueous medium, with a removal efficiency of 95 ± 5% [13]. In addition to their excellent removal efficiency, the magnetic properties of these composites have allowed for the easy separation of adsorbents from water using an external magnet. Zaghlol et al. prepared macroporous PANI/poly(vinyl alcohol) aerogel, which was used as both an adsorbent (with a maximum adsorption capacity reaching 41.2 mg g^−1^) and a reductant for Cr(VI) ions in aqueous solutions [4]. Bober et al. developed conducting and magnetic composite PANI aerogels containing hexaferrite particles, cryopolymerized in the presence of poly(vinyl alcohol), which worked as adsorbents of Reactive Black 5 with a dye removal percentage of 99% after just 4 h [14]. 

PPy, one of the most well-known conducting polymers due to its high electrical conductivity, has easy preparation (it has been synthesized in several forms, e.g., powders, colloids, films, hydrogels, and cryogels), biocompatibility, and excellent environmental stability in ambient conditions [17], and has been widely used as an adsorbent for the removal of Cr(VI) ions from wastewater [5,18,19]. The most desirable PPy absorbents are easily applicable PPy cryogels, hydrogels, and aerogels, which can be effortlessly removed from the solution after the adsorption process is completed [7,20]. Minisy et al. synthesized PPy-nanofibrillated cellulose aerogels, which were three-dimensional (3D), porous, lightweight, and flexible, and showed a 183.6 mg g^−1^ Cr(VI) ion adsorption capacity with a 98% removal efficiency [7]. Li et al. obtained a nanofiber aerogel through the facile polymerization of pyrrole on a 3D cellulose acetate framework, reaching a maximum adsorption capacity of 244.65 mg g^−1^ for Cr(VI) [19]. Gao et al. also produced 3D macroscopic PPy/reduced graphene oxide composite hydrogels, thanks to simultaneous adsorption and chemical reduction, which showed a Cr(VI) ion removal capability of 395 mg g^−1^ [18]. 

In this work, novel PPy aerogels were prepared via the cryopolymerization of pyrrole with the oxidant in the presence of water-soluble polymers (stabilizers), followed by the freeze-drying of PPy cryogels. The benefit of adding the water-soluble polymers to the polymerization mixture at a low temperature manifested itself in the formation of porous, free-standing, conducting PPy cryogels/aerogels, which were easy to handle due to the mechanical properties introduced by the stabilizers. For the first time, the effects of various stabilizing agents (gelatin, (poly(*N*-vinylpyrrolidone) (PVP), including poly(vinyl alcohol) (PVAL), methylcellulose (culminal) or hydroxypropylcellulose (klucel)) on the final properties of PPy cryogels/aerogels were investigated. The choice of stabilizers strongly influenced their morphology, DC conductivity, and mechanical properties. All of the prepared PPy aerogels were applied as adsorbents of Cr(VI) ions from water. The adsorption performance of PPy aerogels on Cr (VI) was consistently studied at different initial concentrations of Cr(VI) ions at different contact times. The adsorption kinetics were evaluated in order to better understand the adsorption mechanism. The kinetics of Cr(VI) ions’ uptake was examined with pseudo-first-order, pseudo-second-order, and intraparticle diffusion models. Additionally, in order to determine the interaction between the adsorbent and the Cr(VI) ions, experimental data were analyzed with adsorption isotherm models (Langmuir, Freundlich, and Temkin models). 

## 2. Results and Discussion

### 2.1. Characterization of Polypyrrole Aerogels

**Figure 1:**
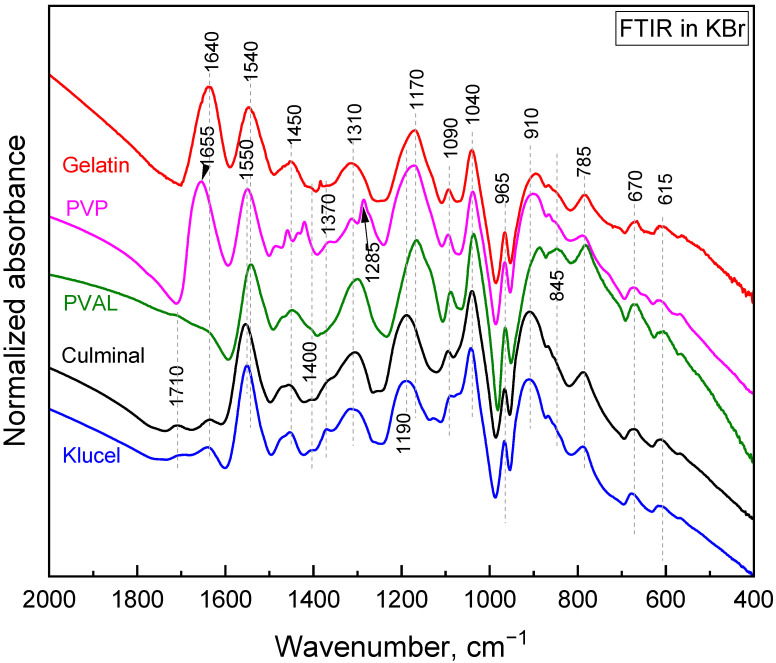
FTIR spectra of the PPy cryogels.

The polymerization of pyrrole in the presence of water-soluble polymers at room temperature led to the formation of colloidal particles [21]. It was observed that the choice of the water-soluble polymer had an impact on the obtained particles’ size and shape. When the same polymerization was carried on in frozen conditions, conducting and porous cryogels were obtained. In the present study, PPy aerogels prepared in the presence of various stabilizing polymers were successfully produced using a facile cryopolymerization technique. The formation of PPy was confirmed with FTIR spectroscopy (Figure 1). The typical bands of PPy, at 1540/1550 cm^−1^ (C–C stretching in pyrrole ring of PPy salt/base), 1450 cm^−1^ (C–N stretching in pyrrole ring), 1310 cm^−1^ (C–H and C–N in-plane deformations), 1170 cm^−1^ (C–H and N–H in-plane deformations in PPy base), 1090 cm^−1^ (N–H^+^ deformation), 1040 cm^−1^ (C–H and N–H in-plane deformations), 965 cm^−1^ (out-of-plane C–C deformations of the pyrrole ring), 910 cm^−1^ (C–H out-of-plane deformations in PPy base), 785 cm^−1^ (C–H out-of-plane deformations), 670 cm^−1^ (out-of-plane C–C deformations of the pyrrole ring and C–H rocking), and 615 cm^−1^ (out-of-plane N–H deformations), were observed in all PPy aerogels [22,23,24]. These spectra corresponded to partially deprotonated PPy and are consistent with previously analyzed PPy cryogels [25]. The stabilizing polymers were reflected in the FTIR spectra as well. Gelatin was manifested, with its carbonyl stretching at 1640 cm^−1^ [26]. PVP displayed a carbonyl stretching band at 1655 cm^−1^, a group of C–H deformation-related bands around 1450 cm^−1^, CH_2_ wagging coupled with C–N stretching at 1285 cm^−1^, and C–C stretching, which contributed as a shoulder of the band at 910 cm^−1^ [26]. PVAL was observed via its C–O stretching vibrations and contributed as a shoulder of the band at 1170 cm^−1^ with its C–C–O stretching at 845 cm^−1^ [26]. Cellulose derivatives showed a weak band of carbonyl stretching (overoxidation) at 1710 cm^−1^, an O–H deformation band at 1640 cm^−1^, C–H deformation bands at 1400 and 1370 cm^−1^, and C–O stretching contributes in the region 820–1150 cm^−1^ (klucel has a defined band at 1130 cm^−1^) [26,27].

**Figure 2:**
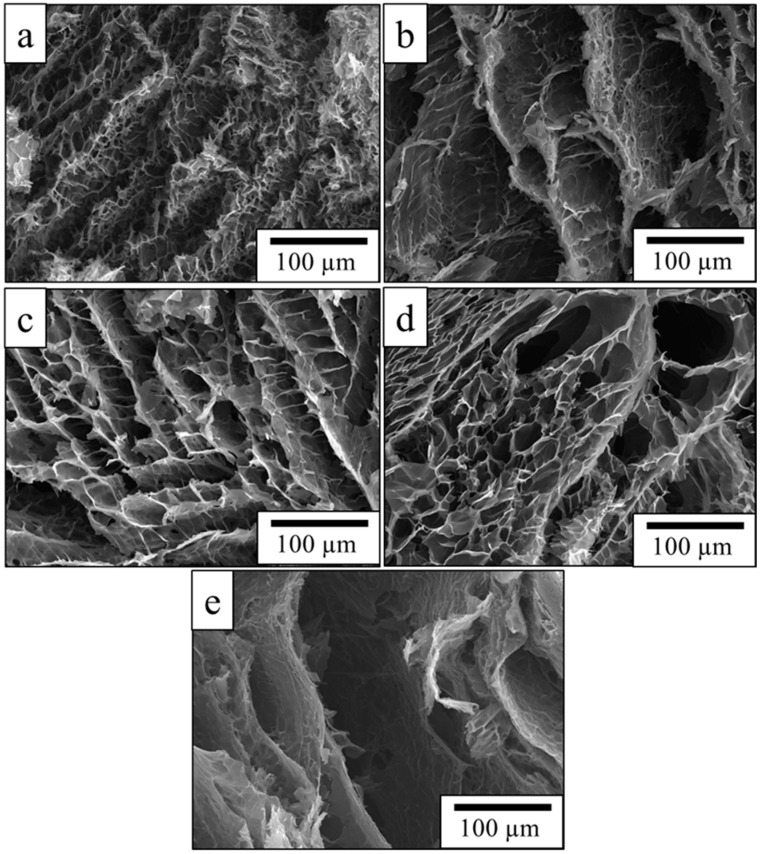
SEM images of PPy aerogels prepared in the presence of (**a**) PVAL, (**b**) PVP, (**c**) klucel, (**d**) gelatin and (**e**) culminal.

Scanning electron microscopy (SEM) revealed that all the PPy aerogels possessed uniform, interconnected 3D macroporous networks (Figure 2); however, similar to the formation of colloidal particles, the sizes of the macropores and micropores could vary through the use of water-soluble polymers. Generally, it is well known that the porous structure of cryogel formed during cryopolymerization is mainly determined by the formation of ice crystals [14]; however, clearly, it can be seen that the nature of the stabilizer not only modified the ice crystal formation rate and size but also influenced the pore structure of the final PPy aerogels (Figure 2). The smallest pores visible by SEM were formed while PVAL (Figure 2a), klucel (Figure 2c), and gelatin (Figure 2d) were used as the stabilizing agents. The pore size was below 100 µm for all three PPy aerogels. On another side, when PVP (Figure 2b) and culminal (Figure 2e) were used as stabilizing agents, much larger pores were formed bigger than 300 µm.

As can be seen from Table 1, the porosity of PPy aerogels obtained after freeze-drying was very similar in all cases (91 to 95%), excluding the PPy-PVP aerogel reaching just 63%. This might be connected to the compact wall structure of PPy-PVP (Figure 2b) and low pore volume (1.3 cm^3^ g^−1^) when compared to the other PPy aerogels (Table 1).

Similarly, the effect of water-soluble polymers (used during cryopolymerization) on the mechanical properties (Young’s modulus, tensile strength, and strain at break) of PPy cryogels could be strongly noticed (Table 1). The Young’s modulus of the PPy cryogels swollen with water was the highest for the PPy-gelatin (461 kPa) and the lowest for PPy-PVAL cryogels (19 kPa) (Table 1). In the case of the PPy cryogel, which was prepared in the presence of a culminal, the mechanical properties were not evaluated due to its unitability in a swollen state. It is worth noticing that PPy-culminal aerogel possessed very poor mechanical properties in its swollen form (cryogel); however, after freeze-drying, it maintained its integrity, and the handling properties were good enough for the investigation of the PPy-culminal aerogel as an adsorbent. 

The DC conductivity values, measured on PPy aerogels compressed to pellets, were also dependent on the water-polymer used for the preparation of the PPy cryogels (Table 1). The highest DC conductivity was found for the PPy-PVAL and PPy-gelatin cryogels, 6.1 and 2.4 S cm^−1^, respectively. This seems to be connected to the morphology. Both PPy-PVAL and PPy-gelatin cryogels had the most uniform network without the presence of large pores (Figure 2a,d). Such morphology guarantees the better connectivity of the conducting PPy phase in the aerogel, which improves the transport of charges and consequently increases the conductivity compared to the PPy aerogels with larger pores. The lowest DC conductivity of 3×10^−2^ S cm^−1^ was obtained for PPy-klucel aerogels. The DC conductivity of PPy aerogels prepared in this study was in good agreement with the conductivity previously reported (10^−2^ to 10^−5^ S cm^−1^) in the literature for similar aerogels [14,20].

### 2.2. Adsorption of Chromium (VI) Ions

#### 2.2.1. Effect of Contact Time

**Figure 3:**
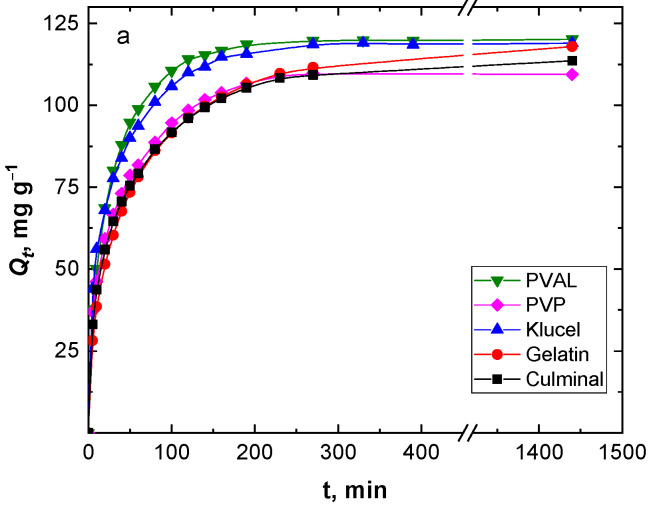

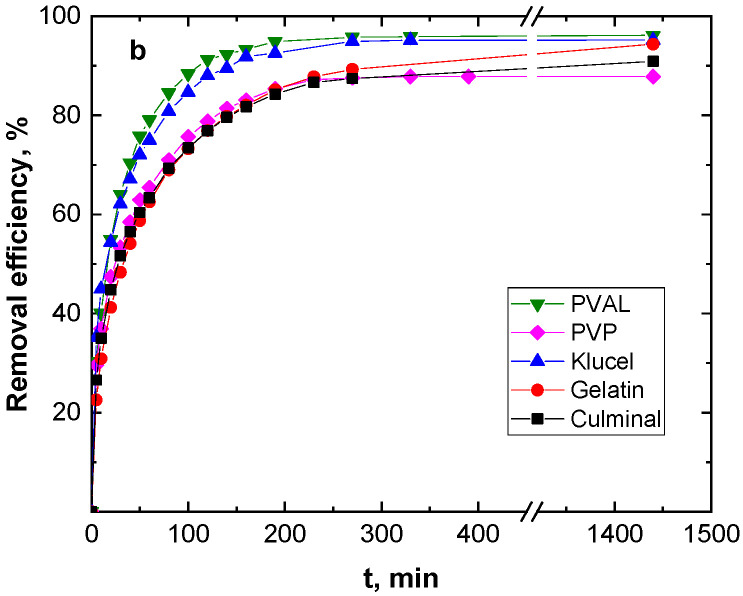
Effect of contact time on the removal capacity (**a**), and removal efficiency (**b**) of Cr(VI) ions (50 mg L^−1^, 25 mL, pH 2) by various PPy aerogels (0.01 g) at room temperature.

Figure 3 shows the effect of the contact time on the removal capacity and efficiency of the Cr(VI) ions by various PPy aerogels. It is clearly visible that the amount of Cr(VI) ions adsorbed onto the PPy aerogels increased with time, and the adsorption rate was much faster at the beginning of the process before gradually slowing down until equilibrium was attained. The fast adsorption at the early stage was due to the availability of the surface binding sites, which were gradually occupied during the adsorption process. Various PPy aerogels showed a similar adsorption behavior toward the Cr(VI) ions. The continuous and smooth *Q*_t_ vs. time adsorption curves predicted a monolayer adsorption process of Cr(VI) ions onto the surface of the PPy aerogels [28]. PPy–PVAL had the highest removal efficiency of 96.2%, while PPy–PVP showed the lowest efficiency at 87.8% removal. This could be explained based on the fact that PPy-PVAL aerogel had the highest porosity while PPy–PVP had the lowest porosity and the smallest pore volume (Table 1). Additionally, PPy–klucel showed 95.2%, PPy–gelatin showed 94.4%, and PPy–culminal showed 90.9% removal efficiencies.

#### 2.2.2. Adsorption Kinetics

**Figure 4:**
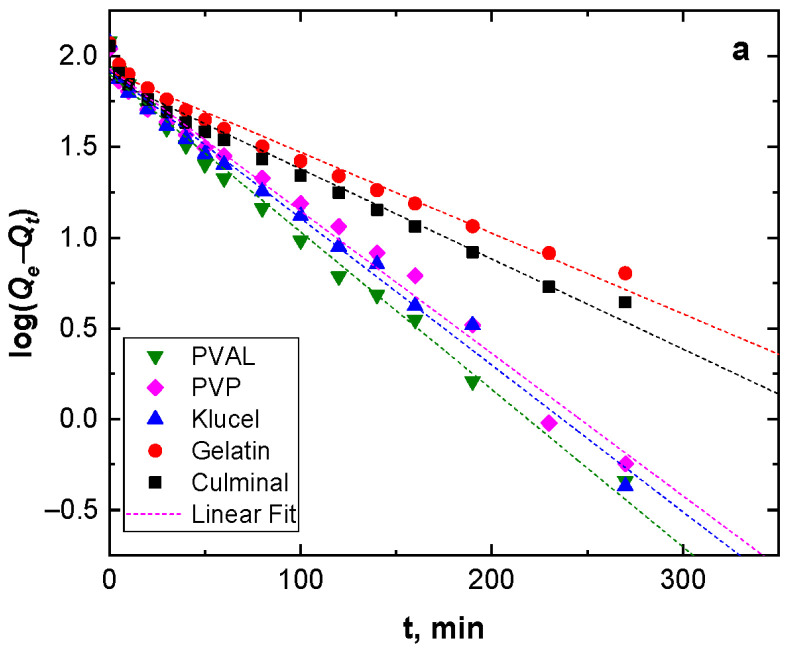

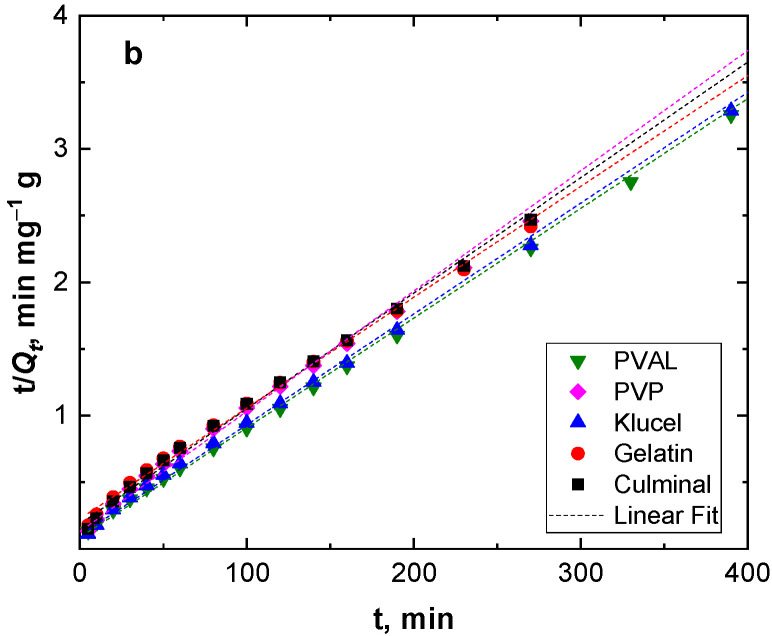
Pseudo-first-order (**a**), and pseudo-second-order (**b**) kinetics modeling.

Adsorption kinetics describe the adsorption rate and the interactions between the adsorbate and the adsorbent (adsorption mechanism). Pseudo-first-order and pseudo-second-order models were used to study the adsorption kinetics (Figure 4). Table 2 presents the kinetic parameters and correlation coefficient (*R*^2^) values. The *R*^2^ values for the pseudo-second-order model were very close to the unit (0.999) for all the PPy aerogels and were always higher than *R*^2^ for the pseudo-second-order model (0.960–0.986). In addition, the calculated equilibrium adsorption capacities of the pseudo-second-order model agreed well with the empirical results. This implies that the adsorption of Cr(VI) ions onto all PPy aerogels followed pseudo-second-order kinetics, indicating that the adsorption of Cr(VI) ions onto the PPy aerogels was controlled by chemisorption behavior. This chemisorption behavior might be due to the electrostatic interaction and anion exchange process between the counter-ions (Cl^−^) and HCrO^4−^ ions. The obtained data were in good agreement with the previously published results of Cr(VI) ions adsorption onto PPy composites [29,30].

#### 2.2.3. Intraparticle Diffusion

**Figure 5:**
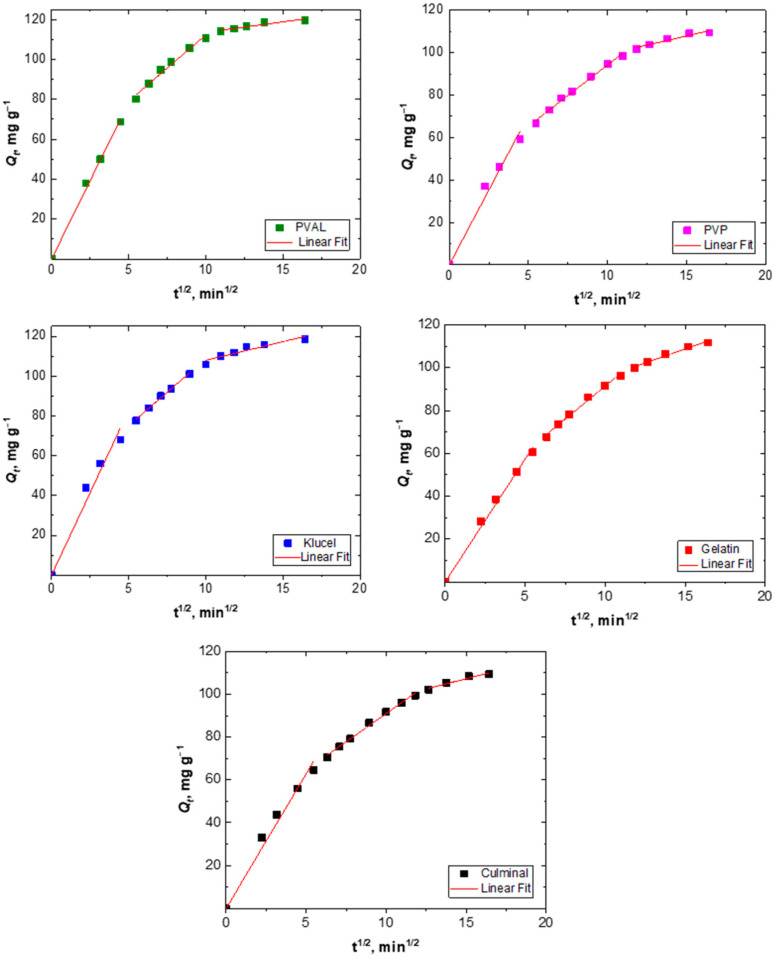
Intraparticle diffusion modeling of Cr(VI) removal onto PPy aerogels.

The adsorption process of a solute into a porous adsorbent was controlled by three types of mechanisms; surface diffusion onto the external surface of the adsorbents, intraparticle diffusion into the interior of pores of adsorbents, and the sorption of the adsorbate onto the interior binding sites of the adsorbent [31]. The adsorption of Cr(VI) ions onto the PPy aerogels was analyzed using the intraparticle diffusion model by plotting *Q_t_* versus *t*^1/2^ (Figure 5) in order to find out if intraparticle diffusion was the rate-limiting step of the adsorption process. The plots of all PPy aerogels showed multi-linearity (Figure 4), which indicated that the adsorption of Cr(VI) took three stages to process [32]. The first stage is attributed to external surface adsorption, while the second stage describes the gradual diffusion process, where intraparticle diffusion is rate-limiting, and the third stage is attributed to the slow final equilibrium process. The intraparticle diffusion rate constant (*k*_i_) and the *c* values were determined from the slope and the intercept of the second linear sections (Table 2). The higher the intercept (*c*) value, the bigger the role of interparticle diffusion was as a rate-limiting step. Among all the prepared PPy aerogels, PPy–PVAL was the one onto which the adsorption process was most controlled by intraparticle diffusion (with the highest *c* value of 45.9 mg g^−1^).

#### 2.2.4. Effect of Initial Cr(VI) Concentration

**Figure 6:**
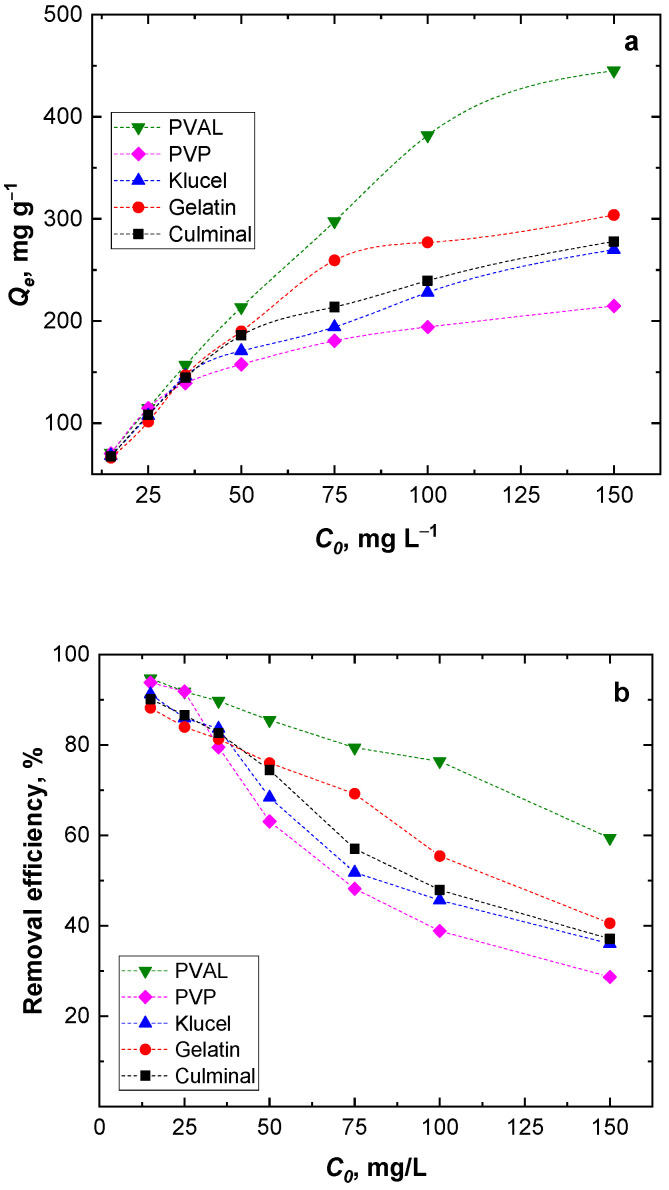
Effect of the initial Cr(VI) ions concentration on the equilibrium capacity (**a**) and removal efficiency (**b**).

The amount of Cr(VI) adsorbed onto the PPy aerogels (*Q_e_,* mg g^−1^) was found to increase with the increasing Cr(VI) ions’ initial concentration (Figure 6a). However, by increasing the Cr(VI) initial concentration, the removal efficiency decreased (Figure 6b). At higher Cr(VI) ion concentrations, the available binding sites on the surface of the PPy aerogels saturated fast, and within a short time, no more available free binding sites for all Cr(VI) ions present in the solution were available. Among the aerogels, PPy-PVAL always had the highest removal efficiency, while PPy-PVP had the lowest removal efficiency, especially at high Cr(VI) initial concentrations (Figure 6). This, again, could be attributed to the lowest porosity, pore size, and volume of the PPy-PVP aerogel (Table 1), which could be directly connected to the lowest specific surface area.

#### 2.2.5. Equilibrium Adsorption Isotherms

**Figure 7:**
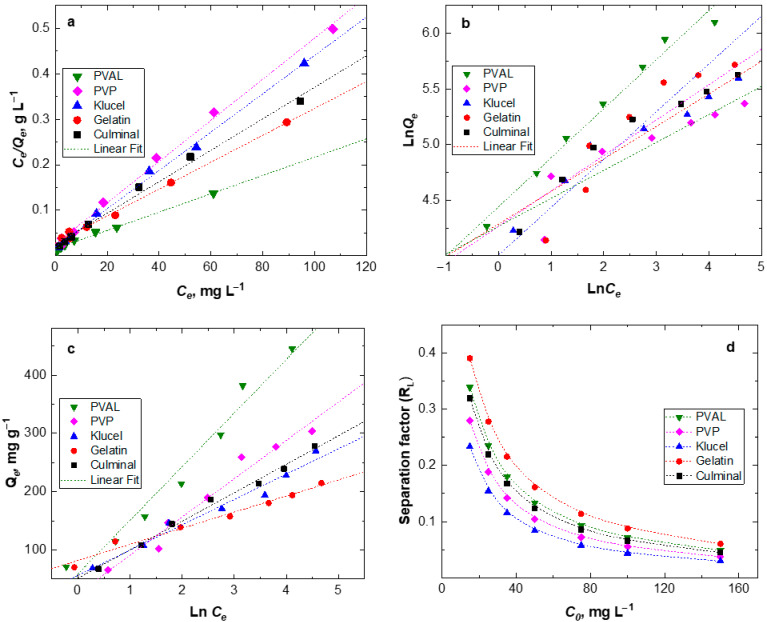
Equilibrium adsorption isotherms of Cr(VI) onto various PPy aerogels; Langmuir (**a**), Freundlich (**b**), Temkin (**c**) and Langmuir separation factor (**d**) modelling.

To estimate the maximum adsorption capacity of the various PPy aerogels for the removal of Cr(VI) ions from aqueous solutions, the equilibrium isotherms were studied (Figure 7). The Langmuir isotherm describes the homogenous monolayer adsorption of chromium ions onto the surface of the adsorbents. The Freundlich isotherm expresses heterogeneous multilayer adsorption, and the Temkin isotherm describes the heat of adsorption due to the adsorbate (metal ions) and adsorbent (PPy aerogels) interactions [28]. The calculated parameters of isotherms and linear coefficient *R*^2^ are listed in Table 3. The results show that the Langmuir isotherm model has the highest *R*^2^ values for all PPy aerogels, implying that the adsorption of Cr(VI) ions onto the PPy aerogels is better described by the Langmuir model than by other models. This indicates that the adsorption took place as a homogenous monolayer on the surfaces of PPy aerogels. The calculated maximum adsorption capacities are presented in Table 3. PPy–PVAL has the ultimate adsorption capacity of 497.5 mg g^−1^, which was relatively high compared to the majority of PPy-based adsorbents (Table 4), as previously reported in the literature [7,29,30,33]. Minisy et al. obtained the adsorption capacities toward Cr(VI) ions with the highest value of 183.6 mg g^−1^ onto PPy– nanofibrillated cellulose aerogels [7]. Kera et al., by using the PPy–PANI/iron oxide nanocomposite as an adsorbent, reached an adsorption capacity of 303 mg g^−1^ at pH 2 [29]. Zhang et al. prepared a uniform fiber ball loaded with PPy, which showed an adsorption capacity of 86.74 mg g^−1^ [30]. Choe et al. synthesized the PPy nanotube, which accomplished maximum adsorption of 205.34 mg g^−1^ [34]. Hosseinkhani et al. coated the surface of sulfonated cellulose fibers with PPy, and such composites showed a maximum adsorption capacity of 198 mg g^−1^ [35]. Shao et al. prepared the PPy/bacterial cellulose composites with a nanofiber structure, which possessed a maximum adsorption capacity of 555.6 mg g^−1^ at pH 2 [36]. The highest adsorption capacity of PPy-PVAL aerogels could be attributed to the highest porosity (95.2%) and pore volume (15.4 cm^3^ g^−1^) (Table 1) among all the investigated PPy aerogels. The Freundlich model predicted a favorable adsorption process for all the PPy aerogels with the values of 1/*n* < 1. The divergence of the slope from 0.5 indicated that intraparticle diffusion partially affected the rate-limiting step, besides other processes controlling the overall adsorption process [37]. The greater the 1/*n* value, the higher the favorability of adsorption. PPy–PVAL showed the most favorable process (Table 3). The small values of heat for the adsorption-related parameter (*B*), as obtained from the Temkin model, indicated that there were weak interactions between the Cr(VI) ions and PPy chains through the electrostatic interaction or ion-exchange mechanisms [38].

Further analysis of the Langmuir isotherm could be performed based on a dimensionless equilibrium parameter called separation factor *(R_L_*), which is defined as follows:

$$R_{L}=1/(1+K_{L}C_{i})$$

The *R_L_* value indicates the nature of the adsorption process: unfavorable (*R_L_* > 1), linear (*R_L_* = 1), favorable (0 < *R_L_* < 1), or irreversible (*R_L_* = 0). *K_L_* is the Langmuir constant, and *C_i_* is the initial concentration of Cr(VI) ions. A lower *R_L_* value indicated a more favorable adsorption process. Figure 7d shows that all *R_L_* values for all the PPy aerogels were in the range of 0.02 to 0.4, which provided an indication that the adsorption of Cr(VI) onto PPy aerogels is a highly favorable process. Additionally, *R_L_* was found to decrease by increasing the Cr(VI) ions’ initial concentrations. PPy–klucel aerogels had the lowest *R_L_* values among the PPy aerogels, which implied their highest affinity toward the Cr(VI) uptake, while PPy–gelatin had the highest *R_L_* values and the lowest affinity toward Cr(VI) ions. 

#### 2.2.6. FTIR Spectroscopy of PPy Aerogels after Adsorption of Cr(VI) Ions

**Figure 8:**
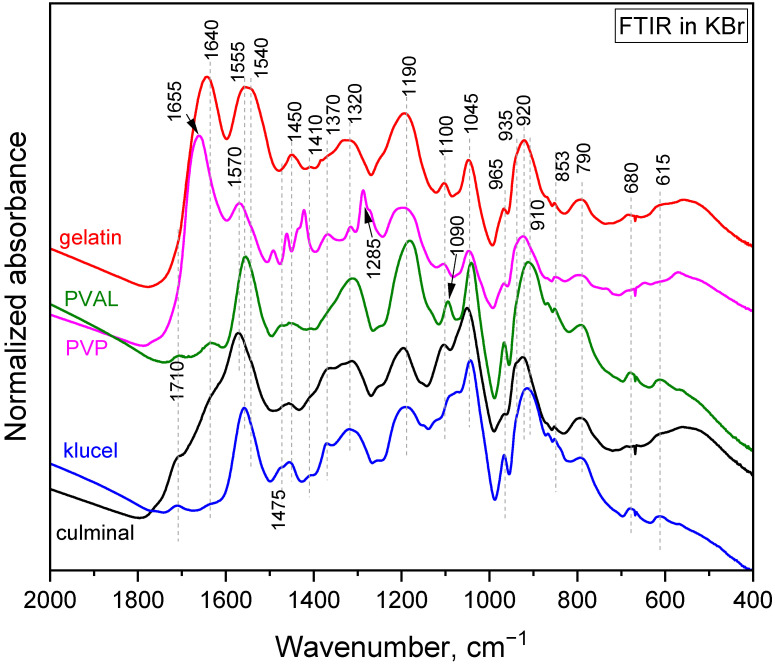
FTIR-Cr. FTIR spectra of PPy cryogels with adsorbed Cr(VI).

**Figure 9:**
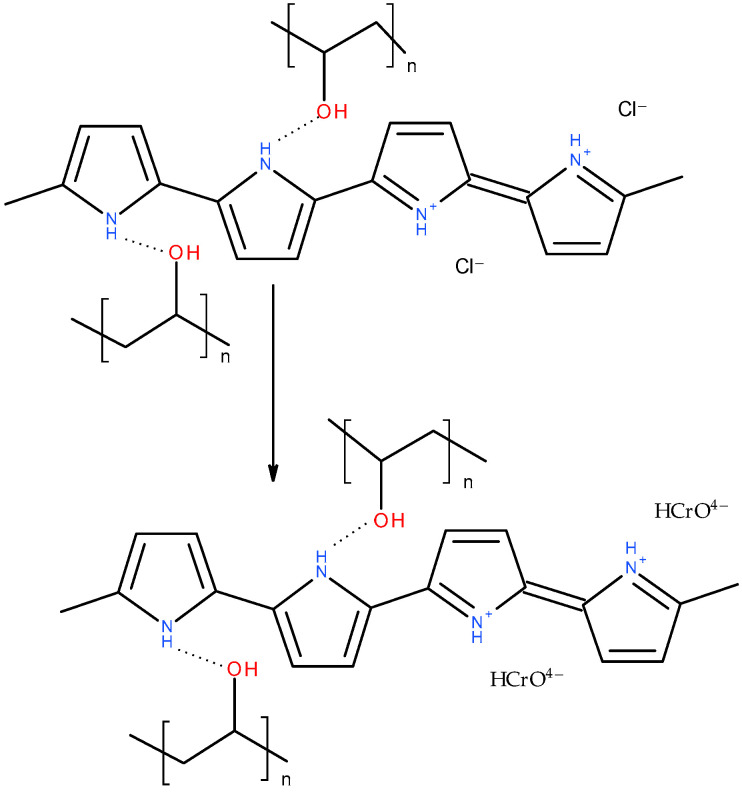
Adsorption mechanism of Cr(VI) ions onto PPy–PVAL aerogel.

After the adsorption of Cr(IV) (Figure 8), the pyrrole ring C–C stretching band shifted to 1550 or even 1570 cm^−1^, the pyrrole ring C–N stretching band formed a maximum at 1475 cm^−1^, C–H or C–N in the in-plane deformation band shifted to 1320 cm^−1^, the pyrrole ring breathing band shifted to 1190 cm^−1^, the N–H^+^ deformation shifted to 1100 and decreased in intensity, the C–H and N–H in-plane deformation band shifted to 1045, the band out of plane C–C deformations of the pyrrole ring decreased, the PPy out-of-plane C–H deformation band shifted to 920 cm^−1^ with a shoulder at 935 cm^−1^, and the C–H out-of-plane deformation band shifted to 790 cm^−1^. These changes corresponded to the decreased protonation of PPy [22,23]. They are more or less pronounced in all the aerogels except the one stabilized with klucel, which was less protonated from the beginning. Additionally, a small peak appeared at 853 cm^−1^. This band, together with a band at 905 cm^−1^, which was not observed probably due to an overlap with a PPy C–H deformation, could be attributed to HCrO^4−^, which was adsorbed by an electrostatic (outer–sphere) interaction [41,42]. Other forms of chromium were not detected with FTIR spectroscopy. Based on the adsorption kinetic study and FTIR spectroscopy, the proposed adsorption mechanism is presented in Figure 9. 

## 3. Conclusions

Mechanically stable, porous, and conducting PPy cryogels/aerogels were obtained by the cryopolymerization of pyrrole in the presence of various water-soluble polymers in a frozen state. These obtained physicochemical properties could be easily varied by the choice of the stabilizer used. The highest DC conductivity (6 S cm^−1^), porosity (95%) and pore volume (15 cm^3^ g^−1^) were found for the PPy-PVAL aerogel. While the PPy-PVP aerogel had the lowest porosity (63%), and pore volume (1.3 cm^3^ g^−1^). The best mechanical properties were possessed by the PPy-gelatin aerogel, which reached Young’s modulus of 461 kPa and tensile strength of 32.7 KPa. All the PPy aerogels were applied as adsorbents of Cr(VI) ions from an aqueous solution, and the adsorption efficiency was investigated in detail. The adsorption process followed pseudo-second-order kinetics for all the PPy aerogels, which indicates that the adsorption was controlled by chemisorption behavior. PPy–PVAL possessed an adsorption capacity of 497.5 mg g^−1^ for the removal of Cr(VI) ions from the aqueous solution and a removal efficiency of 96.2%, pointing to the high potential of such materials as efficient adsorbents in water-pollution treatment. Additionally, such porous and conducting materials could also be applied as electrode materials for energy conversion and storage applications, as well as in biological applications. 

## 4. Materials and Methods

### 4.1. Preparation of Polypyrrole Aerogels

Polypyrrole cryogels were synthesized by the oxidation of pyrrole (0.2 M, 98%, Sigma-Aldrich, Shanghai, China) with iron(III) chloride hexahydrate (0.5 M, ≥99%, Sigma-Aldrich, Schnelldorf, Germany) as an oxidant in the presence of 6 wt% aqueous solutions of various steric stabilizers (poly(vinyl alcohol) (Mowiol^®^ 10-98, Sigma-Aldrich, Schnelldorf, Germany), poly(*N*-vinylpyrrolidone) (PVP; type K-60, Fluka, Epalinges, Switzerland), hydroxypropylcellulose (Klucel, type 99-EF, Hercules, Aqualon Division, Wilmington, DE, USA), gelatin (porcine type, Fluka, Epalinges, Switzerland), and methylcellulose (Culminal MC, Ashland, OR, USA). The oxidant/pyrrole mole ratio was 2.5 in the final polymerization mixture. The monomer and oxidant were dissolved separately in the aqueous solution of the stabilizer before being quickly mixed by shaking and immediately sucked into a plastic syringe, frozen in solid carbon dioxide/ethanol suspension (−78 °C) for 30 min, and kept in a freezer (−24 °C) for one week. After the pyrrole cryopolymerization was complete, the PPy cryogels were thawed at room temperature (24 ± 1 °C). Afterward, they were removed from syringes and immersed in an excess of 0.2 M hydrochloric acid to remove any remaining reactants and by-products. The corresponding PPy aerogels were prepared by the freeze-drying (GREGOR Instruments, L4-110 freeze-drier, Czech Republic) of cryogels.

### 4.2. Characterization

PPy formation was confirmed by Fourier transform infrared spectroscopy (FTIR) in region 4000–400 cm^−1^ using a Thermo Nicolet NEXUS 870 FTIR Spectrometer (DTGS TEC detector; 64 scans; resolution 2 cm^−1^) in a transmission mode on samples that were ground to potassium bromide pellets. The spectra were corrected for the carbon dioxide and humidity in the optical path.

PPy aerogels morphology was investigated by the scanning electron microscope (SEM) Vega Plus TS 5135 (Tescan, Brno, Czech Republic). 

Static mechanical properties of PPy cryogels (diameter 3 mm; length 60 mm) in the deionized water at room temperature were studied with an electromechanical testing device Instron 6025/5800R (Instron, Norwood, MA, USA) equipped with a 10 N load cell and with a cross-head speed of 10 mm min^−1^. 

DC electrical conductivity was evaluated by a Van der Pauw method on pellets (diameter 13 mm; thickness ≈1 mm) made of compressed PPy aerogels at 530 MPa by a hydraulic press Trystom H-62 (Olomouc, Czech Republic). The pellets were inserted in a home-made sample holder and equipped with four gold-plated spring-loaded equidistant electrodes that made contact at the boundary of the pellet. A Keithley 230 Programmable Voltage Source was connected serially with a Keithley 196 System DMM, which supplied the current: a Keithley 617 Programmable Electrometer was used to estimate the potential difference. The value is averaged from the measurements in two perpendicular directions (room temperature; relative humidity 35 ± 5%). To avoid heat dissipation in the sample, the current was kept below 1 mA.

An internal porous structure of PPy aerogels was measured on a mercury porosimeter Pascal 140 and 440 (Thermo Finigan, Rodano, Italy) which operated in two pressure intervals, 0–400 kPa and 1–400 MPa, and enabled the determination of pore size from 0.004 to 116 µm. Program Pascal by means of Washburn’s equation using a cylindrical pore model was used to calculate the pore volume and the most frequent pore diameter. Porosity (*p*) was gained by the formula:

*p* = (*V* × 100)/(*V* + 1/ρ) (%)

where *V* is the cumulative pore volume, and ρ is the sample density.

### 4.3. Removal of Hexavalent Chromium Ions 

For a typical adsorption experiment, 10 mg of the PPy aerogel was immersed in 25 mL of Cr(VI) aqueous solution (50 mg L^−1^) under mild stirring (150 rpm) at room temperature (20 ± 2 °C) and pH 2. Cr(VI) removal was followed by a UV-Vis measurement (Lambda 950 spectrometer, Perkin Elmer, Coventry, UK).

Adsorption capacity, *Q_e_* (mg g^−1^), and removal efficiency, *R* (%), were calculated according to the equations:

$$Q_{e}=\frac{\left(C_{i}-C_{e}\right)}{m}\times V$$

$$R\%=\frac{\left(C_{i}-C_{e}\right)}{C_{i}}\times 100$$

where *C_i_* and *C_e_* (mg L^−1^) are the initial and equilibrium concentrations of Cr(VI), respectively, *V* (L) is the volume of the Cr(VI) solution and *m* (g) is the mass of the aerogel.

The kinetics of Cr(VI) ions’ uptake was analyzed using pseudo-first-order, pseudo-second-order, and intraparticle diffusion models as follows:

Pseudo-first-order:

$$\log(Q_{e}-Q_{t})={\mathrm{logQ}}_{e}-\frac{k_{1}}{2.303}t$$

Pseudo-second-order:

$$\frac{t}{Q_{t}}=\frac{1}{k_{2}Q_{e}^{2}}+\frac{t}{Q_{e}}$$

Intraparticle diffusion:

$$Q_{t}=k_{i}t^{\frac{1}{2}}+c$$

where *Q_e_* and *Q_t_* (mg g^−1^) are the quantity of Cr(VI) ions’ uptake at equilibrium and time *t* (min), respectively, *k*_1_ (min^−1^), *k*_2_ (g mg^−1^ min^−1^), and *k*_i_ (mg g^−1^ min^−1/2^) are the pseudo-first-order, pseudo-second-order, and intraparticle diffusion kinetic rate constants, respectively, and *c* is the intraparticle diffusion constant. 

The equilibrium adsorption isotherms were studied by varying the initial concentration of the Cr(VI) solution (15–150 mg L^−1^), which was brought into contact with the PPy aerogel (5 mg) at pH 2 under mild stirring (200 rpm) until equilibrium was achieved (24 h) at room temperature. Langmuir, Freundlich, and Temkin models were used to explore the experimental data as follows:

Langmuir

$$\frac{C_{e}}{Q_{e}}=\frac{1}{Q_{\max}K_{L}}+\frac{C_{e}}{Q_{max}}$$

Freundlich

$${\mathrm{lnQ}}_{e}={\mathrm{lnK}}_{F}+\frac{1}{n}{lnC}_{e}$$

Temkin

$$Q_{e}=B{\ln K}_{T}+B\ln C_{e}$$

where *Q*_max_ (mg g^−1^) is the maximum adsorption capacity at saturation, *K_L_*, *K_F,_* and *K_T_* are constants in the Langmuir, Freundlich, and Temkin isotherm models, respectively, and *n* is a constant in relation to the heterogeneity of the adsorption. Adsorption is favorable for 0.1 < 1/*n* < 1 (the bigger the 1/*n*, the higher the favorability). *B* is a Temkin constant in relation to the heat of adsorption. The models’ validity was confirmed by a correlation with the experimental data and the best fit of the kinetic and isotherm models was predicted based on the linear regression coefficient (*R*^2^) value.

## Figures and Tables

**Table 1:** Mechanical properties of polypyrrole cryogels, DC conductivity, pore diameter, volume, and porosity of corresponding freeze-dried PPy aerogels.

Stabilizer	Young’s Modulus, kPa	Tensile Strength, kPa	Tensile Strain at Break, %	DC Conductivity, S cm^−1^	Pore Diameter, µm	Pore Volume, cm^3^ g^−1^	Porosity, %
PVAL	19	1.5	9.3	6.1	10.4	15.4	95.2
PVP	78	6.3	12	3 × 10^−1^	0.6	1.3	63.2
Klucel	54	2.8	7.7	3 × 10^−2^	23.6	13.7	94.7
Gelatin	461	32.7	14.8	2.4	7.8	12.7	94.3
Culminal	mechanically unstable in a swollen state			1.5 × 10^−1^	21.1	7.8	91.0

**Table 2:** Pseudo-first-order, pseudo-second-order and intraparticle diffusion adsorption kinetic parameters.

Stabilizer	*Q_e_* (exp), mg g^−1^	Pseudo First-Order			Pseudo Second-Order			Intraparticle Diffusion		
		*Q_e_*_,_ mg g^−1^	*k*_1,_ min^−1^	*R* ^2^	*Q_e_*, mg g^−1^	*k*_2_, g mg^−1^ min^−1^	*R* ^2^	*c,* mg g^−1^	*k*_i_, mg g^−1^ min^−1^	*R* ^2^
PVAL	120.2	79.4	0.020	0.989	121.5	7.9 × 10^−4^	0.999	45.9	6.64	0.977
PVP	109.8	85.4	0.018	0.986	110.9	6.2 × 10^−4^	0.999	36.6	5.76	0.992
Klucel	119.1	82.2	0.019	0.987	120.3	6.9 × 10^−4^	0.999	41.5	6.73	0.994
Gelatin	118.0	82.4	0.010	0.975	120.3	3.1 × 10^−4^	0.999	29.9	6.15	0.998
Culminal	113.6	75.1	0.011	0.977	115.5	4.0 × 10^−4^	0.999	40.2	5.27	0.993

**Table 3:** Equilibrium isotherm parameters.

Stabilizer	Langmuir			Freundlich			Temkin		
	*Q*_max_, mg g^−1^	*K_L_*, L mg^−1^	*R* ^2^	*K_F_,* mg g^−1^	1/*n*	*R* ^2^	*K_T_*, L g^−1^	*B*	*R* ^2^
PVAL	497.5	0.130	0.992	84.89	0.44	0.981	1.90	92.03	0.969
PVP	221.2	0.172	0.996	71.19	0.25	0.815	1.93	27.69	0.979
Klucel	237.5	0.219	0.997	72.69	0.29	0.952	3.63	43.56	0.972
Gelatin	337.8	0.104	0.996	54.98	0.43	0.876	1.47	65.71	0.965
Culminal	289.0	0.142	0.993	71.20	0.32	0.944	2.86	48.96	0.969

**Table 4:** Cr(VI) ion adsorption capacity of various materials based on PPy reported in the literature.

Adsorbent	Conditions	Adsorption Capacity (mg g^−1^)	Ref.
Fiber ball/PPy	pH 2; 50 °C	86.74	[30]
PPy– nanofibrillated cellulose aerogels	pH 2; RT	183.6	[7]
Cellulose sulfate fibers/PPy	pH 2	198	[35]
PPy nanotube	pH 2; 25–45 °C	119.3–205.3	[34]
PPy–PANI/iron oxide nanocomposite	pH 2; 25 °C	303	[29]
PPy/ cellulose acetate aerogels	pH 2; 25–45 °C	182.8–322.6	[19]
PPy/ chitosan aerogel	pH 2	401	[39]
TEPA/PPy/GO aerogel	pH 2; 25 °C	408.5	[40]
PPy aerogels	pH 2; RT	221.2–497.5	This work
PPy/bacterial cellulose composites	pH 2; 25 °C	555.6	[36]

## Data Availability

Data are available upon request.
